# Comparing Methods of Detecting an Elusive Dasyurid Marsupial, the Threatened Julia Creek Dunnart (*Sminthopsis douglasi*), in Central Western Queensland, Australia

**DOI:** 10.1002/ece3.70507

**Published:** 2024-11-04

**Authors:** Alice H. Bakker, Pia Schoenefuss, Greg Mifsud, Susan Fuller, Andrew M. Baker

**Affiliations:** ^1^ School of Biology and Environmental Science Queensland University of Technology Brisbane Queensland Australia; ^2^ Greg Mifsud Consulting Toowoomba Queensland Australia; ^3^ Biodiversity and Geosciences Program, Queensland Museum South Brisbane Queensland Australia

**Keywords:** camera trap, carnivorous marsupial, Dasyuridae, detection methods, live trapping, small mammal, thermal imagery

## Abstract

The Julia Creek dunnart, *Sminthopsis douglasi*, is a small, threatened carnivorous marsupial occurring in scattered populations in the grasslands of central and northwestern Queensland, Australia. The distribution of the species is largely unknown due to sporadic survey efforts and its historically low detection using traditional live trapping methods. There is an urgent need to determine the best methods of detection to optimise survey methodologies and more effectively manage species conservation efforts. In this study, we compared the effectiveness of live (Elliott) traps, baited white flash camera traps and thermal imagery binocular surveying for detecting *S. douglasi*. We deployed 40 white flash camera traps at two sites in Bladensburg National Park (south of Winton), where the species is known to occur, for three consecutive periods between June and November 2022. Four comparative sessions of live trapping were undertaken between April and August 2022 at the same locations. During the live trapping periods, a total of 12 nights of surveying were conducted with thermal imagery binoculars in a preliminary assessment of the technique. The total live trapping effort was 3600 trap nights (approximately 700 trap nights per site in each trapping event). Live trapping resulted in 12 detections of individual *S. douglasi* from 19 total captures. The highest trap success on a given trapping session was 1.71%, and overall trap success from both sites across all sessions was 0.53%. In comparison, baited camera traps (deployed facing the ground at 70 cm range) took 1,269,884 images over 5383 trap nights. There were 11 confirmed images of *S. douglasi*, on three individual occasions, which represented 2.10% of all small mammal captures and just 0.0009% of the total images. Four species of small mammals were detected using camera traps, whereas live trapping detected only two species. No small mammals were detected on any of the 12 thermal binocular surveys. Overall, our study highlights the comparative high utility of traditional live trapping for detecting *S. douglasi*. This research provides a framework for ongoing monitoring of the Bladensburg National Park population. It will be more broadly beneficial for informing the best detection techniques of *S. douglasi* in ongoing work investigating the overall distribution of the species. Similar studies assessing multiple detection methods for small terrestrial mammals have shown an advantage of white flash camera traps compared to other traditional detection techniques. Our contrasting results serve as a reminder that the utility of different techniques for detecting small mammals is best assessed on a species‐by‐species basis.

## Graphical abstract

The Julia Creek dunnart, *Sminthopsis douglasi*, is a small, threatened carnivorous marsupial occurring in scattered populations in the grasslands of central and northwestern Queensland, Australia. In this study, we compared the effectiveness of live (Elliott) traps, baited white flash camera traps and thermal imagery binocular surveying, for detecting *S. douglasi*. Our study highlights the comparative high utility of traditional live trapping for detecting this threatened dasyurid marsupial.

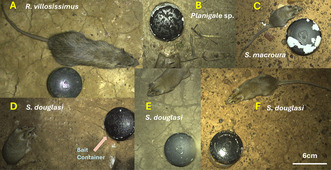

## 1 Introduction

Earth's biosphere is imperilled by human activities, and some of the most iconic fauna threatened with extinction are mammals (Ricketts et al. 2005). Australia has a highly diverse and endemic mammal fauna (Short and Smith 1994) and the worst record for modern mammal extinctions anywhere on Earth (Woinarski et al. 2019). Moreover, Australia has a high diversity of small, terrestrial mammals, which are often elusive and cryptic, posing monitoring challenges for ecologists and conservation managers (Woinarski, Burbidge, and Harrison 2015; Geyle et al. 2020). For such species, a critical element in their management is an effective method of detection (Draper, Marques, and Iriondo 2019). It is essential to be able to detect threatened species both rapidly and confidently, to monitor population trends, identify locations for conservation priority or plan future land‐use change for farming or development (Martin, Kitchens, and Hines 2007; Garrard et al. 2015; Legge et al. 2018). Importantly, even if considerable time and effort is invested in the study of rare and threatened species, the use of unreliable detection methods can produce erroneous estimates of conservation status, habitat requirements and population trends (Tyre et al. 2003; Elphick 2008; Reid et al. 2013), which leads to poor conservation outcomes.

In recent decades, new methods and technologies for detecting small, cryptic mammals have increased in popularity (Vine et al. 2009; Paull, Claridge, and Cunningham 2012; Diggins et al. 2016). Today, two of the most widely used trapping methods are traditional live trapping using metal box traps (e.g., Elliotts or Shermans) and camera traps (e.g., see Tasker and Dickman 2001; How and Cooper 2002; Baker et al. 2003; González‐Esteban, Villate, and Irizar 2004; Flowerdew et al. 2004; De Bondi et al. 2010; Decher, Norris, and Fahr 2010; Lazenby, Mooney, and Dickman 2015; McDonald et al. 2015; Hohnen et al. 2019). These methods have been widely used due to historic effectiveness, the range of data that can be obtained, and their affordability (González‐Esteban, Villate, and Irizar 2004; Stanley and Royle 2005; Wiewel, Clark, and Sovada 2007; De Bondi et al. 2010). Depending on the study objectives, camera traps may be the most appropriate method of detection as they can be used to estimate population size, species richness, site occupancy or relative abundance indices and activity budgets (Nichols, Karanth, and O'Connell 2011). However, it can be difficult to identify individuals within a species (and in some cases, the species itself) in small mammal studies (Palencia et al. 2021). They also generate a large number of images that require many hours of manual identification and sorting and are difficult to store (Schneider et al. 2020). Live trapping, while more labour intensive for the researchers and invasive for the animal (Weldy et al. 2019), provides an opportunity to gather a variety of data unobtainable from camera trap studies, such as morphometrics, size and growth data, tissue samples for genetic assays or voucher specimens (De Bondi et al. 2010). When the species lacks visually identifiable markings, a major benefit of live capture versus camera traps is that the former is a more reliable way of identifying and monitoring individual animals through time using a mark‐recapture method (Paez, Sundaram, and Willoughby 2021). The mark‐recapture method allows for more reliable population estimates and monitoring of population changes for hard to identify species (e.g., Parsons, Orloff, and Prugh 2021; Bakker et al. 2024).

More recently, thermal imagery technology has been trialled for potential use as a detection method for cryptic mammal species that may be otherwise disturbed or less detectable using other methods (Augusteyn, Pople, and Rich 2020; Karp 2020; Underwood, Derhè, and Jacups 2022). Thermal imagery detection methods have proven successful in mammal research on various species, with studies utilising line (i.e., camera, mobile) or point (i.e., binocular, stationary) transect distance sampling approaches (Augusteyn, Rich, and Hemson 2018; Augusteyn, Pople, and Rich 2020; Vinson, Johnson, and Mikac 2020; McGregor et al. 2021; Underwood, Derhè, and Jacups 2022). Although these studies indicate the technique has promise, further research is required to assess its efficacy, particularly on small species with historically low detectability.

There is still much research needed to determine the combined effectiveness of such detection methods for small, cryptic mammals in Australia, particularly in arid and semi‐arid landscapes. Small mammals that are elusive and have an uncertain abundance and distribution, such as the threatened Julia Creek dunnart (*Sminthopsis douglasi*) from central and northwestern Queensland, have proven historically challenging to detect using traditional live trapping (Mifsud 1999, 2000). The grasslands where the species potentially occurs are extensive, and therefore, it is necessary to determine the best combination of detection techniques before monitoring and conservation programs are scaled up. *Sminthopsis douglasi* is currently listed as Vulnerable in Queensland (Nature Conservation Act; Queensland Government 1992), Vulnerable under Australian federal legislation (EPBC Act 1999) and Near Threatened on the IUCN Red List (IUCN 2019). The species is subject to a range of threats, such as land‐use modification chiefly for cropping and livestock production, predation from feral animals including domestic cats (*Felis catus*) and European red foxes (*Vulpes vulpes*) and climate change (Mifsud 1999). Thus, an urgent evaluation of the best combination of methods to detect this species is required. This will determine the detection methodology for monitoring response to threats and efficacy of conservation measures in *S. douglasi*.

Detection methods used in the past for *S. douglasi* have been limited to live, metal box (Elliott) traps (Mifsud 1999, 2001a, 2001b; Rich 2009, 2011; Baker 2013; Bakker et al. 2024), pitfall traps (Mifsud 1994), spotlighting and thermal camera surveys (Augusteyn, Rich, and Hemson 2018). While Elliott traps have returned sporadic captures of the species, pitfall traps, spotlighting and thermal camera surveys all returned low or no detections of *S. douglasi* (Mifsud 1994; Augusteyn, Rich, and Hemson 2018). Pitfall traps proved extremely difficult to install due to the characteristics of the soil in Bladensburg National Park, which transitions to a cement‐like consistency at a shallow depth (Mifsud 1994). Pitfall traps were installed to the industry standard at the time but were significantly less effective than Elliott traps, and it is plausible that some captured *S. douglasi* escaped the pitfalls (by jumping out) (Mifsud 1994). Meanwhile, thermal cameras have successfully been used to detect *S. douglasi*, albeit in very low numbers (Augusteyn, Rich, and Hemson 2018).

Based on historical research detecting other small mammal species in the Australian landscape (Augusteyn, Rich, and Hemson 2018; Augusteyn, Pople, and Rich 2020; Thomas et al. 2020; McGregor et al. 2021), camera traps and thermal imagery binoculars have the potential to be successful methods of detection for *S. douglasi*. To date, these methods have not been assessed for the species. Therefore, the purpose of the present study was to compare the efficacy of live capture traps, white flash camera traps and thermal imagery binoculars for detecting *S. douglasi*. Our study assessed which detection method was most effective for detecting *S. douglasi* based on four live mark‐recapture surveys, 6 months of camera trapping and 12 nights of thermal binocular surveys. This study was undertaken at Bladensburg National Park, which is the highest‐priority conservation area for the species. The specific aims of this research were to compare detection methods for small mammals at Bladensburg National Park, with a focus on *S. douglasi*, and determine the minimum number of trap nights required for each method to detect *S. douglasi* with high confidence. We also conducted a preliminary comparison on the cost‐effectiveness of different methods that positively detected *S. douglasi*. Based on the success of cameras used in previous studies on small mammals (De Bondi et al. 2010; Welbourne et al. 2015; Thomas et al. 2020), we predicted that remote white‐flash cameras would offer a viable and effective alternative technique to live trapping for detecting *S. douglasi*.

## 2 Methods

### 2.1 Study Site

**FIGURE 1:**
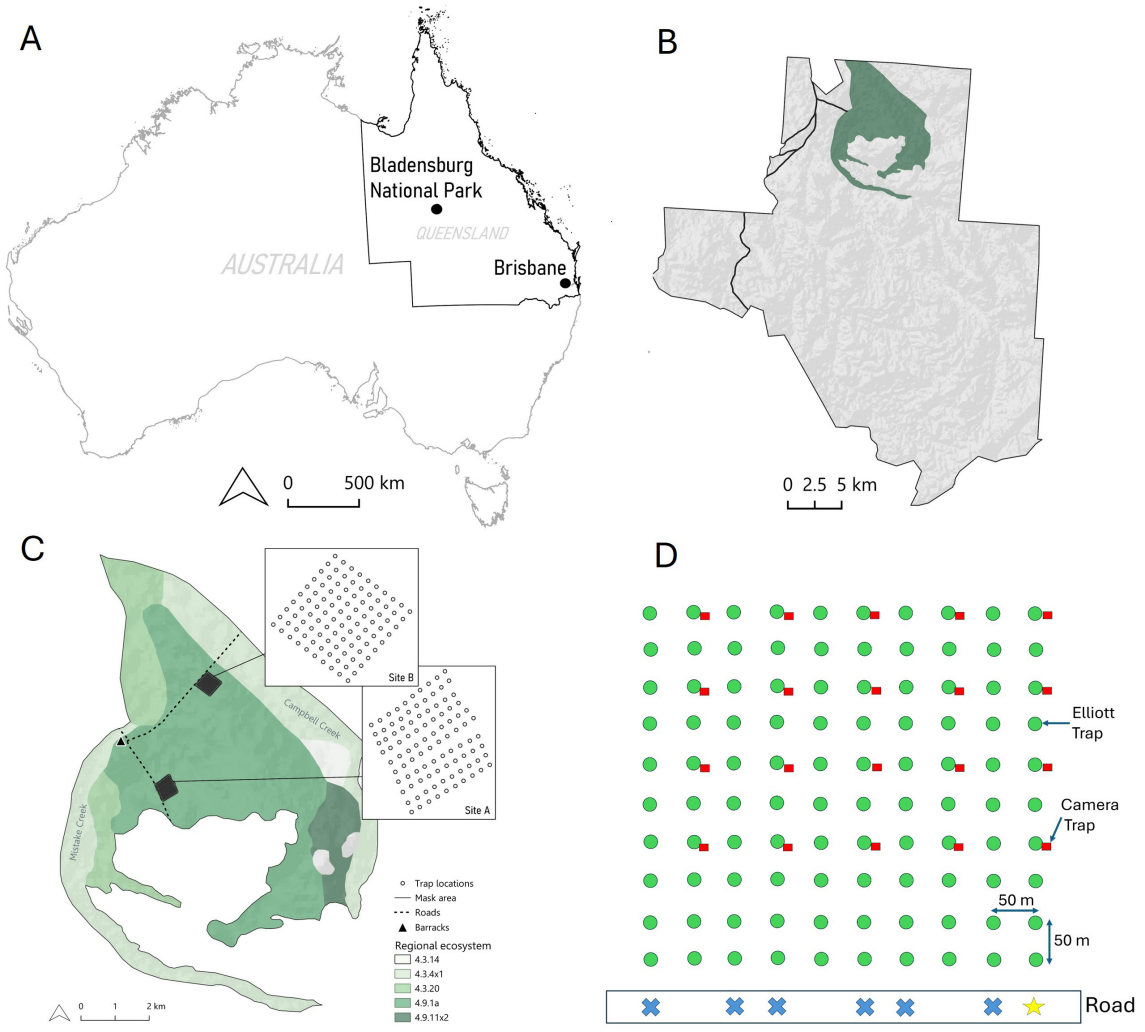
(A) The location of Bladensburg National Park, ~1147 km northwest of Queensland's capital city, Brisbane. (B) Bladensburg National Park boundary, with the only appropriate habitat in the park to support *Sminthopsis douglasi* in the northern section of the park shaded green (adapted from Bakker et al. 2024). (C) The location of study Sites A and B inside Bladensburg National Park with associated Regional Ecosystems, defined from the ‘Biodiversity status of 2021 remnant regional ecosystems—Queensland series v.13 shapefile’. The boundary displayed is a habitat mask of appropriate habitat for *S. douglasi*, defined from the Regional Ecosystems (adapted from Bakker et al. 2024). (D) Schematic drawing showing the trapping grid layout at Sites A and B. The green dots represent live trap locations, and the red squares represent camera trap locations. The black rectangle at the bottom of the figure represents the road. The yellow star symbol shows the starting point of thermal binocular surveys, and the blue crosses show the other six thermal binocular survey points. Green dots (live trap locations) are 50 m apart, and thus, the grid of 100 (10 × 10) green dots measures 450 × 450 m.

Our study was conducted at two sites (denoted Sites A and B) in Bladensburg National Park ~20 km south of Winton in Queensland, Australia (Figure 1).

The work was conducted in parallel to a mark‐recapture study that estimated the density of *S. douglasi* based on live (Elliott) trapping conducted across 2022 and 2023 at Sites A and B (Bakker et al. 2024). Thus, the live trapping data utilised in the present study was a subset (2022 only) of the 2‐year trapping data presented in Bakker et al. (2024), and the study site details and live trapping methodologies outlined below are adapted from that work.

Bladensburg National Park is located in the Mitchell Grass Downs bioregion and experiences an average annual rainfall of 634 mm (Australian Bureau of Meteorology 2023). Dominant plant species at the study sites included *Astrebla lappacea*, *Astrebla pectinata*, *Aristida* sp., *Eragrostis* sp. and *Iseilema vaginiflorum* (Radford et al. 2001). Site A (‘Scrammy grid’) (22°31.678′ S 143°02.767′ E) was selected based on existing (sporadic) *S. douglasi* trapping effort and records (Mifsud 2001b) (Figure 1). Site B (‘Campbells' grid’) (22°30.103′ S 143°03.510′ E) was chosen as a comparative trapping location on the basis that it occurred in the same Regional Ecosystem (RE4.9.1a) as Site A and within the range of other locations in the park where *S. douglasi* was historically found (Figure 1). Regional Ecosystem 4.9.1a is defined as ‘*Astrebla lappacea* tussock grassland to closed tussock grassland, commonly with *Aristida latifolia* and *Iseilema vaginiflorum*. Emergent *Atalaya hemiglauca* and *Ventilago viminalis* may occur on undulating plains formed from Cretaceous mudstones’ (Queensland Government 2021). Initially, we aimed to include a second location that also had some historical capture records of *S. douglasi*. However, due to consistent rainfall in the early stages of the study (April 2022), access to the second location became impossible (being situated across an ephemeral creek). Thus, Site B was chosen as a proximate alternative location. Based on reported average home range sizes (Mifsud 1999; Woolley 2016a), the two sites (A and B) were selected to be more than 8 ha (2.5 km straight line distance) apart. This maximised independence by reducing the chance of trapping the same individuals at both sites.

### 2.2 Live Trapping

All animal trapping and handling was conducted under the auspices of Queensland Department of Environment, Science and Innovation (DESI) Permit P‐PTUKI‐100171210 and QUT Research Ethics Permit 5154.

Julia Creek dunnarts maybe be captured year‐round and in similar abundance throughout the year (Mifsud 1999, 2000, 2001a, 2001b), but the coolest, driest months (across winter, broadly April–October) are the best to conduct field research on the species. This is because even minimal rainfall (5–10 mm) can make the clay soils of the region boggy and difficult to access, and summer temperatures may exceed 40°C, which can cause unsafe fieldwork conditions (e.g., site inaccessibility, heat stress, exposure to snakes) (Bakker et al. 2024).

Thus, mark‐recapture live trapping was carried out between 21 April 2022 and 28 August 2022 across four sessions at both sites. Trapping was conducted using Elliott‐style (type A) aluminium folding traps (see Mifsud 2001a). Each site consisted of 100 traps laid out in a 10 × 10 grid formation. Each trap was set 50 m apart (as per Mifsud 1999, 2001a), and a 50 m buffer was left between the dirt road and the first trap in each transect line to avoid potential road edge effects that may otherwise influence capture rates (Figure 1). Home ranges in *S. douglasi* may be large (0.25–7.13 ha, Mifsud 1999), and population densities relatively low (0.16–0.38 individuals ha^−1^; Bakker et al. 2024), so the trapping area was larger than typically recommended for targeting small mammals and determined to be most appropriate based on several studies conducted on *S. douglasi* at Bladensburg NP (Bakker et al. 2024; Mifsud 2000, 2001a, 2001b). Trap position was determined by GPS coordinates mapped via Google Maps. Each trapping location was marked with thin, white 1.5 m‐long fibreglass marker posts (topped with pink flagging tape) to indicate the trap position.

Minimum trapping effort recommended to successfully capture *S. douglasi* is seven consecutive nights (Mifsud 2001b), and we aimed to match this in every trapping session. However, due to wet conditions restricting site access, seven consecutive trap nights at each site were not always possible (refer to Table 1). When rain prevented access, the traps were kept closed until the ground had dried out sufficiently to continue trapping (typically between 1 and 4 days). Where possible, a total of 700 trap nights were conducted during each trapping session.

**TABLE 1:** Results of live (Elliott) trapping for *Sminthopsis douglasi* at Sites A and B in Bladensburg National Park during 2022.

Date	Site	No. trap nights	No. captures	No. recaptures	Sex ratio M:F	Age ratio A:J	% Trapping success
21/04/2022	A	100^a^	0 (0)	0	0:0	0:0	0
31/05/2022–07/06/2022	A	700	2 (2)	0	2:0	1:1	0.29
02/06/2022–08/06/2022	B	600	3 (3)	0	1:2	1:2	0.50
11/07/2022–18/07/2022	B	700	0 (0)	0	0:0	0:0	0
13/07/2022–20/07/2022	A	700	7 (12)	5	2:1	9:0	1.71
23/08/2022–28/08/2022	A	500	0 (2)	2	2:0	1:0	0.40
25/08/2022–28/08/2022	B	300	0 (0)	0	0:0	0:0	0
Total		3600	12 (19)	7	7:3	4:1	0.53

*Note:* The no. captures column shows the number of new individuals (total number of individuals) captured.

^a^ 6–8 in. of rain in 48 h prevented access to the site for 7 days, precluding further trapping on this trip.

Each trap was baited with a mixture of peanut butter and raw bacon at an approximate ratio of 980 g of peanut butter and 250 g of bacon (as per Bakker et al. 2024). The bait was rolled into a ball and wrapped in a square of biodegradable toilet paper to minimise mess in the traps and for time efficiency when baiting, following methods described by Mifsud (2001b). When traps were reset before sunset, bait ball integrity was inspected for damage by ants. Baits were replaced if they had dried out or if ants had eaten the majority of the ball. Despite ants accessing the bait in traps, there was no evidence of their interference with trapped mammals. Regardless of bait ball condition, rebaiting of all traps was undertaken on the fourth day of trapping (Mifsud 2001b).

Traps were closed during the day, set late in the afternoon as close to dusk as possible, checked at dawn the following morning, and then cleared and closed (maximum 14‐h duration of open traps). If any individuals of the target species were captured, we took them back to the national park barracks (~2–4 km distance) where our research team was able to process the animals at a secure and cool location. For dunnarts, measurements of the head‐body length, tail width, tail length, testis length (for males), left and right pes (hindfoot) length and left ear length were taken with electronic Mitutoyo callipers, and body weight was measured with a Pesola spring balance. Three dunnart species are known to occur at Bladensburg National Park—*S. douglasi*, the stripe‐faced dunnart (*S. macrour*a) and the fat‐tailed dunnart (*S. crassicaudata*) (Tighe 2022). Thus, to ensure accurate identification, for each dunnart capture, photos were taken of the tail, top and bottom of hind feet, ventral and dorsal hair colouring, front and back of the ear, testis (male) or pouch (female), teeth and facial fur colouring. An ear clip (DNA), scats, cloacal swab and fur clip (odour samples) were collected for use in parallel studies. Reproductive condition was assessed, and age (juvenile or adult) was determined by teeth assessment (i.e., state of third upper premolar teeth—the presence of deciduous P^3^ or undescended adult P^3^ was deemed juvenile; fully descended adult P^3^ was deemed adult) (Woolley 2016b; Bakker et al. 2024). A microchip with a passive integrated transponder (PIT) tag (Microchips Australia—Trovan ID‐162B [1.4] ISO FDX‐B Midichip) was inserted into the scruff of the neck for recapture identification. The dunnarts were held at the park barracks during the day until dusk, at which time they were released within 10 m of their point of capture. Individuals of non‐target (i.e., non‐dasyurid) species were identified and immediately released at the point of capture.

### 2.3 Camera Trapping Design

Camera trapping was conducted between June and November 2022, which allowed us to adopt successive deployments of cameras following live trapping. Camera traps were set at both Sites A and B in the grid formation shown in Figure 1. The camera traps were a combination of two Reconyx white flash models: Reconyx Hyperfire 1 HC550—White Flash Camera and Reconyx HP2W Hyperfire 2. These models are essentially equivalent in specifications and operation, with the latter being the newer model from Reconyx. White flash (rather than Infrared) cameras were used because fur colour can be an important factor when distinguishing small mammal species (Thomas et al. 2020). The cameras were deployed at the study sites in fixed positions for three consecutive deployments. The first two camera deployments ranged from 33 to 34 days (8 June–11 July 2022; 20 July–23 August 2022), and the third deployment ranged from 73 to 74 days (28 August–10 November 2022). The third camera deployment was longer due to variations in the timing of field trips necessitated by wet weather conditions. Camera traps were armed and baited at the conclusion of each live trapping survey, and cameras were turned off and bait removed before the commencement of the subsequent live trapping survey. This was to mitigate any potential interference with the accuracy and integrity of detection results.

The cameras were evenly spaced across the pre‐existing grid used for the live trapping, excluding the first 250 m from the road to reduce the likelihood of public interference with the cameras. Camera trap density was determined based predominantly on the coverage of the live‐trapping grid. Live traps were positioned 50 m apart, which was the most appropriate spacing determined by studies on *S. douglasi* home range and density at Bladensburg NP (Bakker et al. 2024; Mifsud 1999, 2001a). Thus, camera traps were spaced at double this distance, given the cameras were being deployed for a longer time period and because we sought an even coverage of the 20 cameras available per site. The cameras were screwed onto a star picket, in a downward‐facing position (Gray, Dennis, and Baker 2017), with the front of the camera 70 cm from the base of a bait container staked into the ground (Thomas et al. 2020). No drift fences were used. The cameras were pre‐set to a focal length of 70 cm, and the detection grid was adjusted by Reconyx to match the focal length. The bait containers were constructed using 60 × 75 mm PVC vent cowls (Bunnings, Australia) and secured to the ground with tent pegs (as per Thomas et al. 2020). The top of each bait container was spray‐painted (flat black colour) to prevent photo under‐exposure. The bait container design allowed the target species to see and smell the bait through the metal mesh, but they were unable to gain access. The bait container lid diameter was 6 cm, which provided a convenient size marker in each photo (Claridge, Barry, and Paull 2010; Gray, Dennis, and Baker 2017; Thomas et al. 2020). Two peanut butter and bacon bait balls (each about 40 mm in diameter) were used per bait container to optimise lasting scent attraction between deployments. These bait balls were replaced between each field trip. Various studies have indicated that the bait remains attractive to small mammals even after 3 months without rebaiting (Thomas et al. 2020; Baker 2021 [unpublished]), so the bait was expected to remain attractive to small mammals throughout the longer third deployment. The cameras were set to maximum sensitivity, with a zero second delay between photos and to take five photos for every motion trigger. The cameras were set to operate 24 h a day due to the cryptic nature of the species in an effort to maximise numbers of detections in case the species might be detected at dawn, dusk or even at times during the day. Colour photos were taken during the day using ambient light and the white flash was only triggered during the night or in low light conditions. Vegetation was cleared in the camera's sensor zone (about a 1 m radius around the bait container) to limit false detections. After each deployment, the bait, SD cards and batteries were collected and replaced.

### 2.4 Thermal Imagery Binoculars

Line transect surveys using Pulsar accolade 2XP50 Pro LRF thermal binoculars (Yukon Advanced Optics Worldwide, Lithuania) were conducted on 12 nights on the roads adjacent to the two trapping grids. These thermal binocular surveys were conducted when the trapping grids were not active with live traps or camera traps. The binocular surveys began approximately 30 min after sundown (as per Augusteyn, Pople, and Rich 2020). The procedure involved driving the car along the road to the first line of traps and using this as the starting point (Figure 1). The first survey was started at this position on the road. The observer with the thermal binoculars stood on the tray of the 4WD utility in an elevated position to view the entire trapping grid. All lights, including car headlights, were turned off, then a timer was started, and the observer would scan the entire trapping grid for a period of 6 min. This protocol had been tested by our field team in preliminary trials and determined to be an appropriate amount of time to scan the site. If a heat signature was detected, the stopwatch would be stopped, and a second fieldworker would walk into the grid with a butterfly net and headtorch following directions via UHF and attempting to catch the animal. The rest of the survey would not resume until all fieldworkers had returned to the car and all lights were turned off. Thirty seconds were allowed to pass before resuming the survey to give animals a chance to resurface if they were disturbed by the capture attempts. This survey was conducted along seven rows for each grid. Six of these rows were established from the road at lines 1, 2, 4, 5, 7 and 8, with a final extended row on line 10 (Figure 1). It is acknowledged that detectability was likely to decrease with distance from the subject due to the possibility that animals were scared away from the road by the car and/or that the vegetation was too thick preventing accurate visuals of heat signatures from a distance. To account for this potential limitation, a longer row on foot was also added on the last line of the grid (line 10). This involved two people starting at the post marked 10‐1 (nearest the road) and walking slowly towards the end of the grid (furthest from the road) for 10 min, with the thermal binoculars in use. Where possible, two nights of surveys at each site were conducted for every live trapping session.

### 2.5 Statistical Analyses

A Fisher's exact test was used to determine any significant association (at *p* < 0.05) between the detection methods and the detection of each species, because assumptions of a chi‐square analysis were not met (Zar 1999; Vieira et al. 2014). Although the numbers of trap nights differed for live trapping and camera trapping, Fisher's test takes this into account. Species were only assessed if they were detected using more than one method.

A binomial distribution was applied to both the live (Elliott) trapping and camera trapping data sets to estimate the probability of a single detection (*pn*) for each species within the overall sample size (*n*). This analysis aimed to determine the minimum required trap nights (*n*) for reliably detecting the presence of a species with a high probability (*pn* > 0.99), following the methodology outlined by Schoenefuss et al. (2024). Total trap nights for Elliott traps were calculated as number of traps multiplied by the number of nights they were open, and camera trap nights were calculated as number of cameras multiplied by the number of nights the cameras were in operation (De Bondi et al. 2010; Swan et al. 2014). Species detected by one method and not the other were excluded from the comparison, due to limited data (e.g., planigales, *Planigale* sp.).

Camera trapping images were identified by eye (A.H. Bakker), and representative photos of each species were corroborated via expert elicitation (A.M. Baker) and with reference to an Australian mammal field guide (Van Dyck, Gynther, and Baker 2013). *Sminthopsis* species were identified by comparisons of size, body shape and fur colour. An exemplar *S. douglasi* individual captured in an Elliott trap and identified based on body weight, hind foot length and fur colouration (refer Bakker et al. 2024) was photographed under a camera trap prior to release, to provide a reference camera photo for the target species. Images were considered to be of a single individual if the series of photos were taken within the same second, and new individuals were counted as a separate detection event if the subsequent image was taken at least 10 min after the previous one (Weerakoon et al. 2014; Gray, Dennis, and Baker 2017).

### 2.6 Cost Comparison

Cost efficiency (in Australian Dollars, AUD) was evaluated by conducting a preliminary cost comparison, encompassing all relevant expenses associated with each detection method (Peres et al. 2017; Mena et al. 2021). This included the purchasing of Elliott traps and camera traps, staff living expenses for field trips, staff wages, hardware for staking out traps and construction of bait containers. In the case of baiting Elliott traps, the cost analysis considered bait ingredients sourced from the most economical products available at Australian supermarkets (as of April 2022). The calculation involved determining the approximate number of bait balls produced during the three field trips in 2022, which was then divided by the cost per 100 bait balls. Fieldwork wages were calculated at $75/day multiplied by a number of staff and number of days. We acknowledge this likely underestimates the real‐life costs for fieldworkers in some studies, but these were the prices applied in the current study, and this cost applies to fieldworkers used for both camera trap and live trap field components, permitting a fair comparison. Four‐wheel drive car hire costs, set at $100/day, were factored in, representing a rate lower than conventional car hire fees but higher than the expenses associated with using a private vehicle (Welbourne et al. 2020). The car hire estimation for each field trip considered 4 days of travel and 7 days of fieldwork for live trapping or 4 days of travel and 2 days of fieldwork for camera trapping. Equipment expenses, such as field guides and animal processing tools, were regarded as in‐kind contributions. We acknowledge the $45/h applied to camera trap image identification work underestimates a paid hourly research assistant rate, but our reduced cost allows for the fact that some of this work may be performed by trained volunteers working at a lower hourly rate.

## 3 Results

### 3.1 Live Trapping

From the 3600 live trap nights conducted in 2022, there were a total of 19 *S. douglasi* captures, with seven recaptures (thus, 12 individuals), representing an overall capture success of 0.53% (Table 1). This equated to one *S. douglasi* capture for every 189 trap nights. Site A had a higher average trapping success for *S. douglasi* of 0.60% compared to Site B, 0.17%. Highest trapping success for Site A occurred between the 13 and 20 July 2022 with a trap success of 1.71%, whereas at Site B, the highest (and only) trapping success was during the trip from 2–8 June 2022, with a trap success of 0.50% (Table 1). In addition to *S. douglasi*, one long‐haired rat (*Rattus villosissimus*) was caught at Site A in early June 2022.

### 3.2 Camera Trapping

**FIGURE 2:**
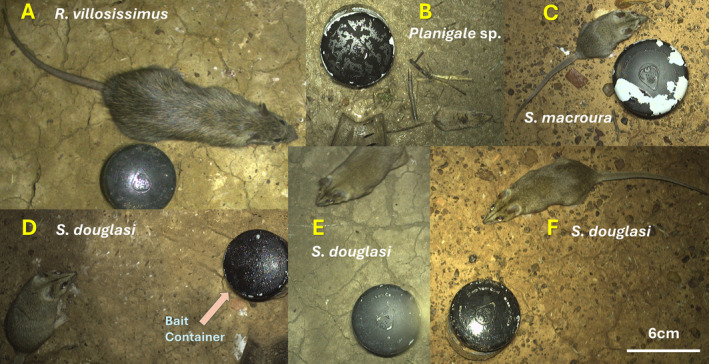
Cropped white flash camera photographs showing all four small mammal species encountered during camera trapping surveys as follows: (A) long‐haired rat (*Rattus villosissimus*), (B) planigale (*Planigale* sp.), (C) striped‐faced dunnart (*Sminthopsis macroura*) and (D–F) Julia Creek dunnart (*Sminthopsis douglasi*). Note the larger size and pelage distinctions (yellowish tinge) of *S. douglasi* (D–F) compared to other dasyurids, *S. macroura* (C) and *Planigale* sp. (B). Note also the larger size, rounded snout and pelage distinctions (shaggy fur) of *R. villosissimus* (A) compared to the dasyurid species (B–F). The diameter of the blackened bait container is 6 cm.

Over the three camera trapping sessions, a total of 1,269,884 images were taken over 5383 trap nights. Each image needed to be manually identified by an observer. Image processing time was approximately 1 s per image, equating to 352.75 h of processing. Of those images, 236 (0.02%) were of small mammal species including *R. villosissimus*, *S. douglasi*, *S. macroura* and *Planigale* sp., as well as several unidentifiable small mammals (due to blurring of the image) (Figure 2). The remaining non‐target vertebrate species recorded were mammals including red kangaroo (*Osphranter rufus*), feral pig (*Sus scrofa*), feral domestic cat (*F. catus*), various reptiles and multiple bird species (species‐level identification of non‐mammalian vertebrates was not attempted). Images of planigales were unable to be identified to species level; thus, they were identified as ‘*Planigale* sp.’ For *S. douglasi*, there were a total of 11 confirmed images, on three individual occasions. Of the four small mammal species, *Planigale* sp. was caught in the highest number, contributing 55.08% of all small mammal captures (Table 2). The second highest total number of small mammal captures was for *R. villosissimus* (33.05% of all small mammal captures), followed by *S. douglasi* (4.66% of all small mammal captures) and lastly *S. macroura* (2.10% of all small mammal captures) (Table 2). *Sminthopsis douglasi* captures represented only 0.0009% of the total camera images.

**TABLE 2:** Summary of white flash camera trapping data from Bladensburg National Park for three deployments between June and November 2022.

Species	Total number of images (individual capture events)	% Of all small mammal captures
*Sminthopsis douglasi*	11 (3)	4. 66
*Sminthopsis macroura*	5 (1)	2.10
*Planigale* sp.	130 (43)	55.08
*Rattus villosissimus*	78 (11)	33.05
*Sminthopsis* sp.	5 (4)	2.11
Unidentified small dasyurid	2 (2)	0.90
Unidentified small mammal	5 (4)	2.10
Total small mammal captures	236 (68)	100

*Note:* ‘capture event’ represents an independent, distinct set of images of a unique individual.

### 3.3 Thermal Imagery Binoculars

Twelve nights of surveying were conducted with the thermal binoculars; seven nights at site A and five nights at site B, due to weather events restricting access to the site. No *S. douglasi* was detected on any of the 12 thermal binocular surveys, and neither was any other species of small mammal. The only mammals detected were the occasional *O. rufus*, but these were distant and not inside the trapping grid, so were not recorded.

### 3.4 Statistical Analyses

The thermal binoculars were not included as a category in Fisher's exact test because they returned zero detections. Only *S. douglasi* and *R. villosissimus* detections were included, as these were the only two mammal species with detections for both live traps and camera traps.

Fisher's exact test was used to assess the significance of the association between the trapping method (Elliott trap or camera trap) and the presence of two species detected using both methods (*S. douglasi* and *R. villosissimus*). The detection method significantly influenced the likelihood of species‐level detection, suggesting that the choice of trapping method was associated with the presence of species. *Sminthopsis douglasi* was significantly more likely to be detected via live trapping (*p* = 0.00), whereas *R. villosissimus* was significantly more likely to be detected via camera traps (*p* = 0.03).

**FIGURE 3:**
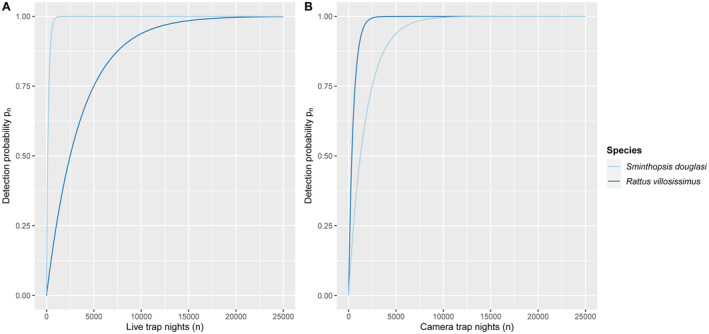
Detection probability (*pn*) of *Sminthopsis douglasi* and *Rattus villosissimus* detected in live (Elliott) traps (A) and camera traps (B) at sites surveyed in Bladensburg National Park.

*Sminthopsis douglasi* was detected more rapidly via live trapping than camera trapping, with near certainty after 871 live trap nights (3600 live trap nights were undertaken) compared to 8261 camera trap nights (5383 camera trap nights were undertaken) (Figure 3; Appendix 1: Table A1). In contrast, *R. villosissimus* was detected more rapidly via camera trapping than live trapping with near certainty after 2252 camera trap nights compared to 16,577 live trap nights (Figure 3; Appendix 1).

### 3.5 Cost Comparison

Human effort and financial cost varied markedly between the two methods, as did the success of the methods for detecting *S. douglasi*. Live trapping was performed at a rate approximately 13‐fold more cost‐effective per detection of *S. douglasi* than camera trapping ($1908.55 and $24,905.67, respectively). Staff wages for live trapping were only 44.23% ($9000) of the cost of camera trapping ($20,373), and overall, the live trapping total cost was 48.58% ($36,262.50) of the cost of camera trapping ($74,717.24) (Table 3).

**TABLE 3:** Preliminary cost comparison to demonstrate cost per detection of *Sminthopsis douglasi* for live (Elliott) trapping (a) and camera trapping (b) at Bladensburg National Park.

(a) Live (Elliott) traps				(b) Camera traps			
Parameter	Quantity	Unit cost	Total cost	Parameter	Quantity	Unit cost	Total cost
**Equipment**				**Equipment**			
Bait ingredients	15	$23.10	$346.50	Bait ingredients	2.4	$23.10	$55.44
Traps	200	$40	$8000	Cameras	40	$1000	$40,000
Fibreglass posts	8	$77	$616	Star pickets	40	$8.50	$340
**Personnel**				Bait containers	40	$10	$400
Fieldwork wages	120	$75	$9000	Attachment bolts	40	$1.22	$48.80
**Travel**				**Personnel**			
4WD hire	15	$100	$1500	Image processing wages	352.74	$45	$15,873
Fuel	28.125	$160	$4500	Fieldwork wages	60	$75	$4500
**Accommodation**	33	$300	$9900	**Travel**			
Food and groceries	3	$800	$2400	4WD hire	33	$100	$3300
**Total cost**			**$36,262.50**	Fuel	28.125	$160	$4500
**Cost per detection of *S. douglasi* (19 total captures)**			**$1908.55**	**Accommodation**	15	$300	$4500
				Food and groceries	3	$400	$1200
				**Total cost**			**$74,717.24**
				**Cost per detection of *S. douglasi* (3 detection events)**			**$24,905.67**

## 4 Discussion

Detection methods used for monitoring rare and elusive species need to be accurate, cost‐effective and time efficient. Small mammals such as the threatened Julia Creek dunnart are notoriously difficult to detect, and their abundance and distribution consequently remain largely unknown. The present study tested whether newer technologies, such as camera traps and thermal imagery, improved detection of *S. douglasi* compared to traditional live‐trapping methods. We showed that live (Elliott) trapping resulted in significantly increased detections of *S. douglasi* compared with white flash camera trapping and thermal binocular protocols. It was predicted based on other small mammal studies that the camera trap orientation (i.e., downwards facing and close range, baited, no drift nets) would provide a viable alternative method of detection for this threatened species. However, despite substantial effort, camera trapping yielded a relatively poor outcome for the target species, although more mammal species were detected using this method (four species) compared to live trapping (two species). The preliminary survey of thermal binoculars yielded no success in detecting *S. douglasi*, or any other small mammal.

Similar studies comparing multiple detection methods for small terrestrial mammals have reported advantage of white flash camera traps and thermal imagery technology compared to other traditional detection methods, which contrasts with the present study. For example, Thomas et al. (2020) found white flash camera traps (baited and set up as per our study) were superior to live (Elliott) traps for detecting small mammals such as the endangered black‐tailed dusky antechinus (*Antechinus arktos*), and common co‐occurring species such as the brown antechinus (*Antechinus stuartii*), fawn‐footed melomys (*Melomys cervinipes*) and bush rat (*Rattus fuscipes*), in forests of eastern Australia. The same was true for Welbourne et al. (2015), who found more detections of all small mammal species in their study using a similar setup for white flash camera traps, including the eastern pygmy possum (*Cercartetus nanus*), brown antechinus, house mouse (*Mus musculus*), bush rat, swamp rat (*Rattus lutreolus*), black rat (*Rattus rattus*) and long‐nosed bandicoot (*Perameles nasuta*). Other studies corroborating the general efficacy of camera traps for mammal detection include those by De Bondi et al. (2010) (agile antechinus [*Antechinus agilis*], yellow‐footed antechinus [*Antechinus flavipes*], mainland dusky antechinus [*Antechinus mimetes insulanus*] and common dunnart [*Sminthopsis murina*]), Zwerts et al. (2021), Wearn and Glover‐Kapfer (2019) (many species), and Clare et al. (2017) (American marten [*Martes americana*]). Similarly, the literature shows a contrasting result to our study when using thermal imaging in a similar way to detect small mammals. Success for this method can be seen in recent studies by Augusteyn, Rich, and Hemson (2018), Augusteyn, Pople, and Rich (2020), Karp (2020), and McGregor et al. (2021).

One study by Hohnen et al. (2019) that examined detection methods for the critically endangered Kangaroo Island dunnart (*Sminthopsis fuliginosa aitkeni*) had much greater success using camera traps set‐up with drift fencing, compared with baited camera trapping or live (Elliott) trapping. It may be hypothesised that dunnart (*Sminthopsis*) species would more likely be captured in pitfalls or on cameras (Bennison, Dickman, and Godfree 2013; Read, Ward, and Moseby 2015; Hohnen et al. 2019; Lavery et al. 2022) because most dunnarts are notoriously shy of live (Elliott) traps (Baker and Dickman 2018). However, this was not the case in our study. Given their verified presence in the live capture surveys across the same trapping grid, *S. douglasi* appeared instead to be relatively shy of the camera traps and, unlike most dunnarts, not reticent to enter (or re‐enter) Elliotts (see also Bakker et al. 2024). It is tempting to speculate that because they are the largest dunnart species (up to 70 g), *S. douglasi* is bolder and exhibits less Elliott trap shyness than congeners. However, this does not explain why they are not also attracted to the baited camera traps.

In our study, both the cameras and the Elliott traps were set up with the same bait, and the general vegetation cover was similar in both cases. A difference in the baiting of the two methods was that while live traps were rebaited approximately every 4 days, camera traps were only baited every field trip, with days between rebaiting ranging from 30 to 72 days. However, during these long bouts between rebaiting, there were still many detections of other small mammal species (notably *Planigale* sp.) throughout the active camera period, suggesting the bait remained attractive.

There are two other potential explanations for our relatively low camera trap detections of *S. douglasi*. First, it is possible that *S. douglasi* individuals were able to distinguish between naturally bare ground around an Elliott trap (depending on its placement) and disturbed bare ground (and perhaps the presence of concentrated animal odours of individuals attracted to the bait) in the cleared zone of the camera traps, where vegetation had been removed to avoid triggering the camera. If so, *S. douglasi* individuals may have chosen to avoid the areas that showed signs of disturbance, concentrated animal presence and/or potential greater exposure to aerial predators (e.g., owls). A second scenario is that the animals were more easily startled by the flash of the cameras (Meek et al. 2014). This is tentatively supported by the fact that in each of the three camera trap detections of *S. douglasi*, there was only 2–5 images in succession of an individual, whereas other sympatric small mammal species, such as *R. villosissimus* or *Planigale* sp., in general stayed under the cameras longer, for up to 10 images across several seconds, and appeared to have a more in‐depth inspection of the bait container. *Sminthopsis douglasi* images were predominantly on the edges of the frame (Figure 2), suggesting that *S. douglasi* were retreating as soon as the flash was triggered. It is therefore plausible that there were more than three detection events of *S. douglasi*, but they were unable to be identified as some images were blurry as the animal moved or was only partially in frame. It is unlikely that this issue was related to the camera detection grid or failure to trigger because the same camera setup and models were used by Thomas et al. (2020) for small mammals and no issues were reported. Moreover, in our study planigales, another dasyurid genus that is much smaller in size and known to be very difficult to capture in live (Elliott) traps (Baker and Dickman 2018), appeared not as easily startled by the flash, having more individual detections, and often remaining under the camera for 5–8 photos and several seconds. Some studies of larger mammal species suggest that animals can become trap shy from white‐flash camera traps (Wegge, Pokheral, and Jnawali 2006). However, there is limited literature on this topic, and it has not been well investigated for small mammal species, especially in an Australian semi‐arid landscape.

Another issue encountered during the camera trapping concerned accurate species identification. Camera trapping resulted in five images of unidentifiable small mammal species, five images that were only identifiable as genus *Sminthopsis*, two images identified only to the family Dasyuridae and 130 images identified as *Planigale* sp. When only parts of an animal were in frame, such as their nose or tail, this led to an assignment as ‘unidentifiable small mammals’ or ‘dasyurids’. Difficulty with the five unidentified images of *Sminthopsis* sp. was due in part to the similar morphology of the dunnart species present in Bladensburg National Park (*S. douglasi*, *S. macroura* and *S. crassicaudata*). Moreover, after consulting several mammal specialists beyond the authorship group, none were able to confidently identify any *Planigale* images to species level, due to the similar morphology of *Planigale ingrami* and *Planigale tenuirostris*, both of which are reported to inhabit the park (Mifsud 1999).

The absence of any small mammal detections using thermal binoculars was surprising, considering the success of other thermal imagery technology used in detecting a range of mammals (Augusteyn, Pople, and Rich 2020; Karp 2020; Vinson, Johnson, and Mikac 2020; McGregor et al. 2021; Underwood, Derhè, and Jacups 2022), including *S. douglasi* in similar vegetation types at a similar distance to the observer (Augusteyn, Rich, and Hemson 2018; Augusteyn, Pople, and Rich 2020; McGregor et al. 2021; Dawlings et al. 2023). There has also been wide success with infra‐red camera traps (Swann et al. 2010; Liu et al. 2013; Wang et al. 2014; Gray, Dennis, and Baker 2017; Hohnen et al. 2019), which share technical similarities with thermal imagery such as detection of heat signatures, night vision detections and are non‐invasive, preventing disruption of natural animal behaviours. The nature of *S. douglasi* and other small mammals seeking shelter in soil cracks on the Mitchell Grass Downs is distinctive (Waudby and Petit 2016), and it seems likely that this sheltering behaviour affects their detectability via thermal imaging—there are abundant holes for refuge. In any case, given the success of similar equipment (Augusteyn, Rich, and Hemson 2018) to detect *S. douglasi*, the utility of thermal imagery for studying this species certainly warrants further investigation.

A clear association between the detection method and trap success was observed in our study. The probability detection curve indicated that a much higher frequency of trap nights is required to detect *S. douglasi* if they are present via the camera trap setup used in this study compared with Elliott traps, and the preliminary cost comparison showed that camera traps also require a substantially larger financial cost per detection. Interestingly, however, our camera trap methodology may be *more* useful than Elliott traps under certain conditions, such as detecting *S. douglasi* during *R. villosissimus* (or other rodent) plagues. For example, there was a *R. villosissimus* plague in 2023/2024 in parts of western Queensland, most likely in response to high rainfall throughout 2023 that continued deep into the summer and early autumn of 2024 (Australian Bureau of Meteorology 2023, 2024). An August–November 2023 study involving live (Elliott) trapping and white flash camera traps (using the same setup adopted here) on two properties 20–50 km south‐east of Julia Creek recorded no *S. douglasi* detections from 1000 live trap nights but captured 700 *R. villosissimus*. And yet, *S. douglasi* was recorded on seven different white flash cameras subsequently deployed at the same sites (Southern Gulf NRM and Baker 2023, unpublished data). Plausibly, the rodents occur in high enough density during a plague such that most live traps are saturated with *R. villosissimus*, but *S. douglasi* may still be detected in low numbers via cameras that were deployed in the 2 months immediately after the live trapping. Similar results were found during April and June of 2024 during the continuing *R. villosissimus* plague via live trapping at Bladensburg National Park, on the same sites where the present work was conducted and *S. douglasi* is known to occur. In this case, there were so many rodent captures that the vast majority of Elliott traps either had captured an *R. villosissimus* individual or were negatively affected by them (trap disturbed, triggered and bait stolen). Under such conditions, the live trapping had much‐reduced efficacy as a detection method for *S. douglasi*, with no individuals captured at Site A, even after 1400 trap nights (Baker 2024, unpublished). Therefore, the general utility of Elliott traps for detecting *S. douglasi* demonstrated in the present study comes with a warning—be aware of the state of flux in co‐occurring rodent densities and choose your detection method(s) accordingly.

In our study, live trapping detected two small mammal species (*S. douglasi* and *R. villosissimus*), whereas the camera traps detected *R. villosissimus*, *S. douglasi*, *S. macroura*, *Planigale* sp. and several unidentifiable small mammals. *Zyzomy argurus* and *S. macroura* have been trapped in Elliott traps in the park in previous surveys (Mifsud 2000, 2001a), and *S. douglasi*, *S. macroura*, *S. crassicaudata*, *Planigale* sp., *Pseudomys desertor*, *R. villosissimus*, *Mus musculus* and *Leggadina forresti* (Tighe 2022) have been detected via camera traps in Bladensburg National Park. Indeed, camera trapping by Tighe (2022) was completed concurrently with our study in 2022 across a range of sites within the same vegetation community surveyed here and showed that camera trapping can be useful for detecting small mammal richness (eight species), highlighting that Elliotts are apparently inferior for this purpose (three species) at Bladensburg National Park. The additional five small mammal species detected by Tighe (2022) were at different sites in the park, so it is possible that localised habitat or environmental differences go some way to explaining the higher species richness. Interestingly, Tighe (2022) detected *S. douglasi* at two sites in 10 photos out of 136,755 images (2284 trap nights), representing 0.0073% of all images, a 10‐fold greater occurrence compared to the present study, where *S. douglasi* images represented only 0.0009% of 1,269,884 images (5383 trap nights). Although Tighe's (2022) camera trap success in detecting *S. douglasi* was higher than our study, it is still low and therefore lends weight to the conclusion that when targeting *S. douglasi* Elliott traps are generally superior to the camera trapping methodology adopted here.

In summary, we have demonstrated the comparative high utility and cost‐effectiveness of traditional live (Elliott) trapping over the adopted camera trapping methodology for detecting *S. douglasi*. Overall, our results stand in contrast to the majority of similar small mammal studies and indeed the favoured methods used to detect many species in the genus *Sminthopsis*. This serves as a reminder that best practice detection for conserving threatened small mammals should where possible be assessed on a case‐by‐case basis.

## Author Contributions

**Alice H. Bakker:** conceptualization (equal), data curation (equal), formal analysis (equal), methodology (equal), writing – original draft (lead), writing – review and editing (equal). **Pia Schoenefuss:** formal analysis (equal), writing – review and editing (equal). **Greg Mifsud:** methodology (equal), writing – review and editing (equal). **Susan Fuller:** supervision (supporting), writing – review and editing (equal). **Andrew M. Baker:** conceptualization (equal), funding acquisition (lead), methodology (equal), project administration (lead), supervision (lead), writing – review and editing (equal).

## Conflicts of Interest

The authors declare no conflicts of interest.

## Data Availability

The authors confirm that the data supporting the findings of this study are available within the article [and/or] its Supporting Information. Code for analyses is available via the Dryad open access repository: https://datadryad.org/stash/share/GqelDq_uCnY4‐ACG‐CewQTmdb01M8FWfKYuJO8AT2J4 and the https://doi.org/10.5061/dryad.n8pk0p34q.

## Appendix 1

**TABLE A1:** (a) Live (Elliott) trap nights (no. trap nights) associated with detection probabilities 0.25, 0.50, 0.75 and 1.00 taken from Figure 3 and (b) Camera trap nights (no. trap nights) associated with detection probabilities 0.25, 0.50, 0.75 and 1.00 taken from Figure 3.

Species	Live (Elliott) trap		Camera trap	
	Detection probability	No. trap nights	Detection probability	No. trap nights
*Sminthopsis douglasi*	0.25	55	0.25	517
	0.50	131	0.50	1244
	0.75	262	0.75	2487
	1	871	1	8261
*Rattus villosissimus*	0.25	1036	0.25	141
	0.50	2495	0.50	339
	0.75	4990	0.75	678
	1	16,577	1	2252
